# Reproductive experience alters the effects of diazepam and fluoxetine on anxiety-like behaviour, fear extinction, and corticosterone levels in female rats

**DOI:** 10.1007/s00213-023-06446-z

**Published:** 2023-08-15

**Authors:** Jodie E. Pestana, Bronwyn M. Graham

**Affiliations:** School of Psychology, University of New South Wales, Sydney, NSW 2052 Australia

**Keywords:** Anxiety, Elevated plus maze, Fear conditioning, Fear extinction, Benzodiazepine, Selective serotonin reuptake inhibitor, Estrous cycle, Corticosterone, Female, Reproductive experience

## Abstract

**Overview:**

Reproductive experience (pregnancy and motherhood) leads to long-term changes in the neurobiological and hormonal features of anxiety in rats and humans. The aim of this study was to examine whether reproductive experience alters the effects of two pharmacological treatments for anxiety, a benzodiazepine (diazepam) and a selective serotonin reuptake inhibitor (fluoxetine), on animal models of anxiety.

**Methods:**

In Experiment 1, virgin (*n* = 47) and age-matched mother (*n* = 50) rats at 1-month post-weaning were injected with diazepam (1.3 mg/kg or 1.7 mg/kg, i.p.) or vehicle, in the proestrus (high estradiol/progesterone/allopregnanolone) or metestrus (low estradiol/progesterone/allopregnanolone) phase of the estrous cycle 30 min prior to the elevated plus maze (EPM). In Experiment 2, virgin (*n* = 25) and mother rats (*n* = 20) were administered fluoxetine (10 mg/kg) or vehicle for 2 weeks prior to being tested on a Pavlovian fear conditioning and extinction protocol, and the EPM.

**Results:**

Replicating past research, in virgin rats, the low dose of diazepam produced anxiolytic-like effects in proestrus, but only the high dose was anxiolytic-like in metestrus. In contrast, in mother rats, both doses of diazepam were anxiolytic-like irrespective of estrous phase. Fluoxetine produced anxiogenic-like effects in virgin rats during fear extinction and the EPM, but had no behavioural effects in mothers. In contrast, fluoxetine increased plasma corticosterone levels measured 30-min post-EPM in mothers, but not virgin rats.

**Conclusions:**

Reproductive experience alters the dose responsivity and efficacy of common anti-anxiety medications in female rats. These findings highlight the importance of considering reproductive status in studies on anxiety and its treatment.

**Supplementary Information:**

The online version contains supplementary material available at 10.1007/s00213-023-06446-z.

## Introduction

Recent research shows that reproductive experience (encompassing pregnancy, lactation, and maternal experience) leads to long-term changes in the behavioural, neurobiological, and hormonal features of anxiety in rats and humans, which persist long after the offspring are weaned (Pestana et al. 2023; Milligan-Saville and Graham 2016; Duarte-Guterman et al. 2019). However, the effects of commonly used treatments for anxiety in reproductively experienced females are largely unknown, with most preclinical studies being conducted in male rodents (Beery and Zucker 2011; Kaluve et al. 2022). Investigating the effects of anxiety treatments in females both with and without reproductive experience is important given that anxiety and stressor-related disorders are twice as prevalent in women compared to men (McLean et al. 2011; Santomauro et al. 2021), and 85% of women will become mothers before the age of 44 (Martinez et al. 2018). Therefore, this study examined the effects of two commonly used pharmacological treatments of anxiety, a benzodiazepine (diazepam) and a selective serotonin reuptake inhibitor (SSRI; fluoxetine), on animal models of anxiety in nulliparous (no reproductive experience) rats and primiparous (one prior reproductive experience) rats.

Although no longer the first-line treatment for anxiety disorders, benzodiazepines are nonetheless a widely used medication (Agarwal and Landon 2019) that produce anxiolytic and sedative effects by acting as positive allosteric modulators of the GABA_A_ receptor (Sigel and Ernst 2018). In nulliparous rats, the estrous cycle (the rodent equivalent of the human menstrual cycle), and the ensuing fluctuations in ovarian steroids estradiol and progesterone (and the progesterone metabolite allopregnanolone, a positive allosteric modulator of the GABA_A_ receptor), influences the sensitivity to benzodiazepines. Whereas a low dose of diazepam produces anxiolytic-like effects during the proestrus (high estradiol/progesterone/allopregnanolone) phase of the estrous cycle, a higher dose of diazepam is required to produce anxiolytic-like effects during the metestrus/diestrus (low estradiol/progesterone/allopregnanolone) phases (Fernandez-Guasti and Picazo 1990; Molina-Hernandez et al. 2001, 2013; Soares-Rachetti Vde et al. 2016). Reduced sensitivity to benzodiazepines during menstrual phases of low ovarian steroids has also been documented in women diagnosed with an anxiety disorder (reproductive status unknown) (Sundstrom et al. 1997). Reproductive experience alters the nature and effects of ovarian steroid fluctuations. For example, reproductive experience reduces the peaks in estradiol across the ovarian cycle in female rats and women (Bernstein et al. 1985; Bridges and Byrnes 2006; Dorgan et al. 1995; Milligan-Saville and Graham 2016), and reduces the peaks in allopregnanolone during proestrus in female rats (Pestana et al. 2023). In addition, we have shown that reproductive experience mitigates the influence of estrous cycle on anxiety-like behaviour (Pestana et al. 2023) and fear extinction (a model of exposure therapy, gold standard treatment for anxiety disorders, Milligan-Saville and Graham 2016) in rats; findings which were translated to women (Pestana et al. 2023; Milligan-Saville and Graham 2016). Given that reproductive experience alters ovarian steroid levels and their effects on anxiety-related tasks, the aim of Experiment 1 was to examine whether reproductive experience also mitigates the influence of estrous cycle on the sensitivity to the anxiolytic-like effects of diazepam.

In Experiment 2, we tested the effects of SSRIs in nulliparous and primiparous female rats. SSRIs are the first line of pharmacological treatment, and require long-term treatment to produce anxiolytic effects (Craske et al. 2017). Unlike benzodiazepines, the anxiolytic-like actions of SSRIs are not reliably detected in animal models of anxiety in males or females (Borsini et al. 2002). In nulliparous female rats, chronic SSRIs have been shown to produce no effect on anxiety-like behaviour (Gray and Hughes 2015; Melo et al. 2012; Sayin et al. 2014; Yohn et al. 2020), anxiolytic-like effects (Yohn et al. 2020), and anxiogenic-like effects (Gray and Hughes 2015). These findings highlight the importance of examining the effects of SSRIs in rodents using multiple models of anxiety and fear (i.e. unlearned anxiety paradigms and fear conditioning/extinction). To date, only one study has examined the effects of SSRIs on fear extinction (the learned inhibition of conditioned fear) in females. This is important given that SSRIs are often prescribed to individuals with anxiety disorders who are also receiving exposure therapy (derived from fear extinction), yet the effects of these combined treatments are largely unknown. Lebrón-Milad et al. (2013) found that chronic fluoxetine (a commonly prescribed SSRI) reduced freezing (a conditioned fear response) during extinction training and extinction recall in nulliparous rats but had no effect in male rats, highlighting the importance of testing the effects of SSRIs in both sexes. However, the effects of SSRIs may be altered in females following reproductive experience, which dynamically modifies the brain’s serotonin system (Lonstein 2019; Pawluski et al. 2019). Indeed, Workman et al. (2016) found that whereas chronic fluoxetine lowered plasma corticosterone and increased neurogenesis in the hippocampus measured 90 min after an acute stressor in nulliparous rats, chronic fluoxetine had no such effects in primiparous rats a few days post-weaning, suggesting that primiparous rats may be resistant to some neurological and endocrinological effects of SSRIs. To our knowledge, no studies have assessed whether the changes to SSRI responsivity following reproductive experience persist beyond weaning once cycling has recommenced, or compared the impact of SSRIs on fear extinction between nulliparous and primiparous rats. As such, the primary aim of Experiment 2 was to investigate whether the effects of chronic fluoxetine on fear extinction differ between nulliparous and primiparous rats (1 month after weaning). This was of particular interest given that reproductive experience alters the neurobiological, hormonal, and behavioural features of fear extinction (Pestana et al. 2021; Tang and Graham 2019b, 2020). A secondary aim of Experiment 2 was to examine whether the effects of chronic fluoxetine on anxiety-like behaviour and the physiological stress response differ between nulliparous and primiparous rats. Combined, the outcomes of Experiment 1 and Experiment 2 suggest that the behavioural and hormonal responsivity to common anxiolytics differ in female rats as a function of reproductive status.

## Methods

### Animals

Experimentally naïve naturally cycling nulliparous and primiparous female Sprague–Dawley rats were obtained from the Animal Resources Centre (ARC), Australia. Breeding in primiparous rats was conducted by ARC prior to their arrival at UNSW. Upon arrival, rats were housed in groups of 5–8 in plastic boxes (67 × 30 × 22 cm) with corncob bedding and a wired lid. The boxes were kept in a 20–22 °C colony room maintained on a 12-h light–dark cycle (lights on 0700 h). Food and water were available ad libitum. Rats remained under these conditions for an acclimatisation period of 2 weeks prior to any procedures. All procedures were approved by the Animal Care and Ethics Committee at UNSW.

### Estrous cycling

Vaginal smears were conducted daily (0900 h–1100 h) to determine estrous cycle phase as previously described (Graham and Daher 2016). A regular 4–5-day estrous cycle consists of four phases: metestrus, diestrus, proestrus, estrus. Vaginal smears began on the first day of handling and continued until the end of each experiment to ensure regular cycling. Handling began 1-month post-weaning in primiparous rats, when their estrous cycle had recommenced (Milligan-Saville and Graham 2016). Nulliparous and primiparous rats were age-matched at the time of each experiment, and underwent behavioural testing at approximately 6 months of age.

### Drug administration

In Experiment 1, rats received an intraperitoneal injection of diazepam (Troy Laboratories Australia; diluted in vehicle: 40% propylene glycol, 10% ethanol, 40% water), or vehicle. Diazepam was administered at a dose of 1.3 mg/kg or 1.7 mg/kg of body weight. Previous studies reported 1.3 mg/kg of diazepam produced anxiolytic-like effects in nulliparous rats during proestrus but not metestrus/diestrus, whereas 2.0 mg/kg produced anxiolytic-like effects during both proestrus and metestrus/diestrus (Fernandez-Guasti and Picazo 1990; Molina-Hernandez et al. 2001, 2013). In our pilot study, many rats fell off the maze after receiving 2.0 mg/kg diazepam, so we used a slightly lower dose of 1.7 mg/kg in the current study. The volume of injection for both doses of diazepam was 1.0 ml/kg of body weight.

In Experiment 2, fluoxetine hydrochloride powder (LKT labs; Sapphire, Bioscience- LKT-F4780) was dissolved in tap water at a daily dose of approximately 10 mg/kg (see Supplemental Information for details). This is the standard dose used to examine the anxiolytic- and antidepressant-like effects of fluoxetine in rodents (Burghardt et al. 2007; Diniz et al. 2022; Graham et al. 2018; Gunduz-Cinar et al. 2016; Norcross et al. 2008), including nulliparous and primiparous female rats (Lebrón-Milad et al. 2013; Workman et al. 2016). The vehicle was tap water. Fluoxetine and vehicle were available ad libitum via the rats’ drinking water bottles, as per Graham et al. (2018). Given that fluoxetine is relatively stable when dissolved in water (Graham et al. 2018; Gunduz-Cinar et al. 2016; Karpova et al. 2011; Lebrón-Milad et al. 2013; Norcross et al. 2008), the water containing the drug was changed every 2 days according to the average weight of the rats and the amount of fluid each box of rats consumed over the 2-day period. To control for any potential effects of handling on behaviour, vehicle-treated rats were weighed on the same days as those receiving fluoxetine. The drug administration procedure continued until all rats had been euthanised.

### Apparatus

#### Elevated plus maze

The elevated plus maze (EPM) apparatus was the same as previously described (arms were 113 cm long × 10.5 cm wide; walls on closed arms were 40 cm high; Pestana et al. 2021). The lighting was 300 l × in Experiment 1, and 160 l × in Experiment 2. These lighting conditions were based on pilot studies in our lab. Video recordings of animal behaviour were scored by the experimenter after behavioural testing was complete.

#### Fear conditioning and extinction

Two sets of experimental chambers served as distinct contexts for conditioning (Context A) and extinction procedures (Context B). These chambers differed in visual and tactile features, as previously described (Graham and Daher 2016). The CS was a 62 dB white noise delivered through a speaker on the wall of each chamber, and the US was a 0.4 mA, 1.0 s footshock delivered through the floor. A computer running Med Associates Med-PC IV controlled presentations of both the CS and the US.

### Procedure

#### Experiment 1

**Fig. 1:**
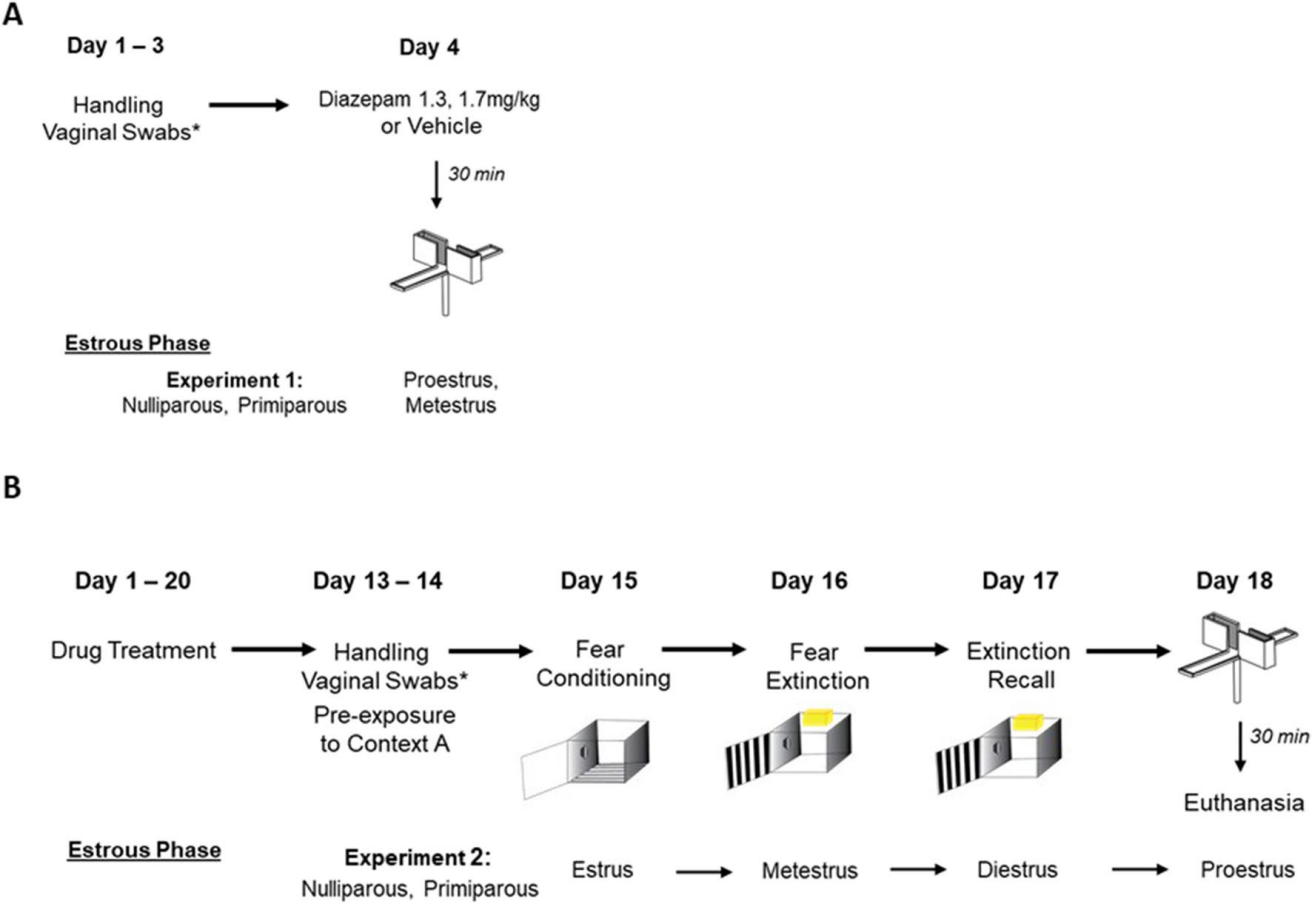
Experiment timeline and estrous phases during each stage of testing in **A** Experiment 1 and **B** Experiment 2

Rats were handled for 5 min each day for three consecutive days (see Fig. 1A). Rats received a single i.p. injection of diazepam or vehicle during either metestrus or proestrus (1200 h–1600 h). Thirty minutes post-injection, rats were placed in the centre of the EPM facing an open arm and observed for a 5-min test (Walf and Frye 2007). The apparatus was cleaned with 70% ethanol in between tests.

#### Experiment 2

##### Handling and pre-exposure

Rats were handled for 5 min for two consecutive days (see Fig. 1B). After handling, rats were individually placed in Context A for 10 min to habituate to Context A.

##### Fear conditioning

Rats were placed in Context A and after a 2-min adaptation period, the CS was presented for 10 s and co-terminated with the shock US. Rats received three CS-US pairings (intertrial interval 85–135 s, with an average of 110 s). Rats were conditioned during estrus (0930–1600).

##### Fear extinction

Rats underwent extinction training 24 h after conditioning in Context B. After a 2-min adaptation period, rats received 30 × 10 s CS presentations, with an intertrial interval of 10 s. Rats were extinguished during metestrus (0930–1600). This is because metestrus is associated with poor extinction recall in nulliparous female rats (Graham and Daher 2016; Graham and Scott 2018; Milad et al. 2009; Milligan-Saville and Graham 2016; Pestana et al. 2021; Rey et al. 2014), and based on Lebrón-Milad et al. (2013), it was predicted that fluoxetine would enhance extinction recall in nulliparous rats.

##### Extinction recall

Rats were returned to Context B 24 h after extinction training. Following a 1-min adaptation period, rats received a single 2-min CS presentation. Rats were tested for extinction recall during diestrus (0930–1600).

##### EPM

Twenty-four hours after extinction recall, rats were tested on the EPM during proestrus (1200–1400 h).

##### Trunk blood collection

Thirty minutes after initiation of the EPM, a subset of rats (8 nulliparous-vehicle rats, 8 nulliparous-fluoxetine rats, 7 primiparous-vehicle rats, 9 primiparous-fluoxetine rats) were euthanised following a brief exposure (45 s) to carbon dioxide (Co2) and decapitated with a guillotine. This timepoint was chosen because plasma corticosterone is elevated following an acute stressor in female rats at this time (Carey et al. 1995; Neumann et al. 1998). Trunk blood was rapidly collected in EDTA-treated tubes (Interpath Services, Victoria, Australia). Blood was centrifuged within 30 min of collection (1000 rcf for 15 min at 4 °C). Plasma was collected and stored at − 80 °C until analysis. All samples underwent one freeze–thaw cycle.

##### Enzyme-linked immunosorbent assay (ELISA)

Plasma samples were diluted in a 50-fold dilution based on pilot studies and previous research (Bekhbat et al. 2018), and tested for corticosterone concentration in duplicates using a commercially available ELISA kit, following manufacturer instructions (KGE009, R&D Systems, USA). Integrated optical density for each sample was measured using an iMARK plate reader (Bio-Rad Laboratories, Hercules, CA) set to a wavelength of at 450 nm. Four-parameter logistic regression software (myassays.com) was used for plotting the standard curve and data extrapolation.

### Scoring

In the EPM, the anxiolytic-like effects of the drugs were manually indexed by a higher number of open-arm entries and a longer time spent in the open arms, whereas the sedative-like effects of the drugs were indexed by a reduced number of closed-arm entries, which reflects general locomotor activity (Fernandes et al. 1999; Pellow et al. 1985). An anxiety index ratio score was calculated and analysed to examine the anxiolytic-like effects of the drugs while taking into account any sedative-like effects on general locomotor activity (Walf and Frye 2007). The anxiety index score was calculated using the following formula: 1 – [(time spent in open arms/test duration) + (open arm entries/total number of open- and closed-arm entries)/2] (Cohen et al. 2013). A higher anxiety index score is indicative of higher anxiety-like behaviour. During scoring, the experimenter was blind to the experimental status of each animal.

Conditioned fear was measured using freezing, defined by the absence of movement except those related to respiration (Fanselow 1980). Rats were manually scored as “freezing” or “not freezing” every 3 s using a time-sampling procedure. A percentage of observed freezing was calculated for each animal to determine the proportion of total observations spent freezing. A second observer blind to experimental group cross-scored a random sample of ~ 30% of conditioned fear data; inter-rater reliability was high (*r* = 0.98, *p* < 0.001).

### Statistical analysis

In Experiment 1, given that the key question of interest was to examine whether the estrous cycle impacts diazepam sensitivity in nulliparous and primiparous rats rather than compare diazepam sensitivity between nulliparous and primiparous rats**,** two-way ANOVAs with the between subject factors of estrous phase (metestrus or proestrus) and drug (vehicle, low dose or high dose of diazepam) were used to assess estrous effects in nulliparous groups and primiparous groups separately. Significant main effects and interactions were analysed using Tukey HSD multiple comparisons, with Games-Howell tests being used if the assumption of homogeneity of variance was violated (according to Levene’s test). One nulliparous rat administered the low dose of diazepam in proestrus was excluded as a statistical outlier (> 2 STDEVs away from the mean on all measures).

In Experiment 2, two-way ANOVAs with the between-subjects factors of reproductive status (nulliparous or primiparous) and drug (fluoxetine or vehicle) were used to assess group differences in pre-CS freezing prior to fear conditioning, extinction training, and extinction recall, as well as CS-elicited freezing during extinction recall. Two-way ANOVAs with repeated measures were used to assess group differences in CS-elicited freezing during fear conditioning and extinction training, with the Greenhouse–Geisser correction used if the assumption of sphericity was violated (as indicated by Mauchly’s test of sphericity). Significant interactions were followed up using independent samples *t-*tests. Planned independent samples *t-*tests with Bonferroni corrections (*p* = 0.025) were used to assess the impact of fluoxetine on extinction recall in nulliparous and primiparous rats separately. One nulliparous-vehicle rat was excluded in the fear conditioning/extinction analysis on the basis that it was a statistical outlier in Block 1 of extinction training (> 6 STDEVs away from the mean), and one nulliparous-fluoxetine rat was excluded on the basis that it was a statistical outlier at extinction recall (> 2 STDEVs away from the mean). Separate two-way ANOVAs with the same between subject factors as above were used to measure anxiety-like behaviour on the EPM, and corticosterone levels measured 30-min post-EPM. Planned independent samples *t-*tests with Bonferroni corrections (*p* = 0.025) were used to assess the impact of fluoxetine on anxiety-like behaviour during the EPM and corticosterone levels in nulliparous and primiparous rats separately. One nulliparous-fluoxetine rat was excluded in the EPM analysis on the basis that it was a statistical outlier (> 2 STDEVs away from the means), and one primiparous-fluoxetine rat was excluded because it fell off the maze during testing. One primiparous-fluoxetine rat was excluded in the corticosterone analysis due to large variability between duplicates (SEM > 300).

## Results

### Experiment 1: Does reproductive experience alter the impact of estrous cycle on diazepam sensitivity?

#### Nulliparous rats

**Fig. 2:**
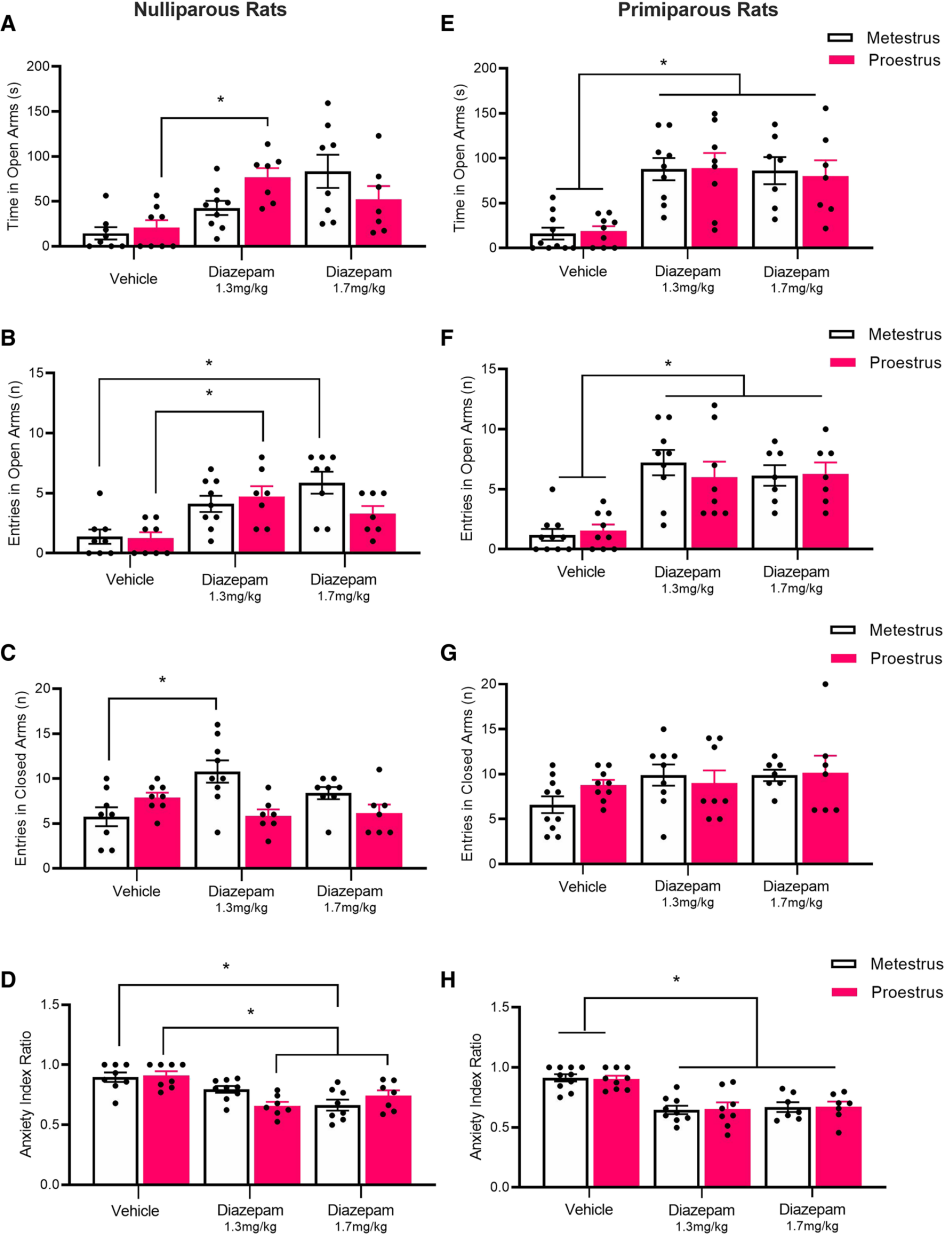
In Experiment 1, nulliparous and primiparous rats in either metestrus or proestrus were administered vehicle, a lower dose (1.3 mg/kg) of diazepam, or a higher dose of diazepam (1.7 mg/kg), and 30 min later were tested on the elevated plus maze. Groups were NP-metestrus-vehicle (*n* = 8), NP-proestrus-vehicle (*n* = 8), NP-metestrus-1.3 mg/kg-diazepam (*n* = 9), NP-proestrus-1.3 mg/kg-diazepam (*n* = 7), NP-metestrus-1.7 mg/kg-diazepam (*n* = 8), NP-proestrus-1.7 mg/kg-diazepam (*n* = 7), PP-metestrus-vehicle (*n* = 10), PP-proestrus-vehicle (*n* = 9), PP-metestrus-1.3 mg/kg-diazepam (*n* = 9), PP-proestrus-1.3 mg/kg-diazepam (*n* = 8), PP-metestrus-1.7 mg/kg-diazepam (*n* = 7), and PP-proestrus-1.7 mg/kg-diazepam (*n* = 7). Note: *ps < 0.05, NP nulliparous, PP primiparous. **A** Mean (± SEM) time spent in the open arms in nulliparous groups. **B** Mean (± SEM) number of entries in the open arms in nulliparous groups. **C** Mean (± SEM) number of entries in the closed arms in nulliparous groups. **D** Mean (± SEM) anxiety index score in nulliparous groups. **E** Mean (± SEM) time spent in the open arms in primiparous groups. **F** Mean (± SEM) number of entries in the open arms in primiparous groups. **G** Mean (± SEM) number of entries in the closed arms in primiparous groups. **H** Mean (± SEM) anxiety index score in primiparous groups. *ps < .05

Figure 2A–D depicts means (± SEM) for behavioural measures on the EPM in nulliparous rats. There was a main effect of drug in the time spent in the open arms (*F*_(2,41)_ = 10.76, *p* < 0.001, ηp2 = 0.34) and number of open-arm entries (*F*_(2,41)_ = 13.50, *p* < 0.001, ηp2 = 0.40), and a main effect of estrous phase in the number of closed-arm entries (*F*_(2,41)_ = 4.91, *p* = 0.03, ηp2 = 0.11). There was an estrous × drug interaction in the time spent in the open arms (*F*_(2,41)_ = 3.87, *p* = 0.03, ηp2 = 0.16) and the number of closed-arm entries (*F*_(2,41)_ = 7.52, *p* < 0.01, ηp2 = 0.27), but no interaction in the number of open-arm entries (*F*_(2,41)_ = 2.70, *p* = 0.08, ηp2 = 0.12). In nulliparous rats tested during proestrus, relative to vehicle, the low dose of diazepam increased the time spent in the open arms (*p* = 0.01, *d* = 2.22) and increased the number of open-arm entries (*p* = 0.02, *d* = 1.83), but had no effect on the number of closed-arm entries (*p* = 0.66, *d* = 1.18). Relative to vehicle, the high dose of diazepam had no effect on the time spent in the open arms (*p* = 0.48, *d* = 0.98), number of open-arm entries (*p* = 0.37, *d* = 1.31), or number of closed-arm entries (*p* = 0.79, *d* = 0.82), during proestrus. In nulliparous rats tested during metestrus, relative to vehicle, the low dose of diazepam increased the number of closed-arm entries (*p* < 0.01, *d* = 1.50) but had no effect on the time spent in the open arms (*p* = 0.13, *d* = 1.31) or number of open-arm entries (*p* = 0.07, *d* = 1.47). In contrast, relative to vehicle, the high dose of diazepam increased the number of open-arm entries during metestrus (*p* < 0.01, *d* = 2.06) and increased the time spent in the open arms, but this did not reach significance (*p* = 0.054, *d* = 1.75), whereas the high dose had no effect on the number of entries into the closed arms (*p* = 0.34, *d* = 1.05). There were no differences between vehicle-treated rats tested during proestrus and metestrus (smallest *p* = 0.57, *d* = 0.90).

In the “anxiety index”, there was a main effect of drug (*F*_(2,41)_ = 16.61, *p* < 0.001, ηp2 = 0.45), and an estrous × drug interaction (*F*_(2,41)_ = 4.12, *p* = 0.02, ηp2 = 0.17), but no main effect of estrous phase (*F*_(1,41)_ = 0.25, *p* = 0.62, ηp2 < 0.01)*.* In nulliparous rats tested during proestrus, relative to vehicle, both the low and high doses of diazepam decreased the anxiety index (*p* < 0.001*, d* = 2.68 and *p* = 0.04*, d* = 1.52, respectively). In contrast, in nulliparous rats tested during metestrus, relative to vehicle, the low dose of diazepam had no effect on the anxiety index (*p* = 0.38, *d* = 1.03), whereas the high dose of diazepam decreased the anxiety index (*p* < 0.001, *d* = 1.92). There were no differences between vehicle-treated rats tested during proestrus and metestrus (*p* = 1.0, *d* = 0.13).

#### Primiparous rats

Figure 2E–H depicts means (± SEM) for behavioural measures on the EPM in primiparous rats. There was a main effect of drug in the time spent in the open arms (*F*_(2,44)_ = 21.81, *p* < 0.001, ηp2 = 0.50) and number of open-arm entries (*F*_(2,44)_ = 23.54, *p* < 0.001, ηp2 = 0.52), but not in the number of closed-arm entries (*F*_(2,44)_ = 2.27, *p* = 0.12, ηp2 = 0.09). There was no main effect of estrous phase or estrous × drug interaction on any measure (largest *F* < 1). In primiparous rats tested during proestrus, relative to vehicle, both the low and high doses of diazepam increased the time spent in the open arms (*p* < 0.01, *d* = 1.96 and *p* = 0.02,* d* = 1.73, respectively) and increased the number of open-arm entries (*p*s < 0.01, *d*s > 1.5), but had no effect on the number of closed-arm entries (smallest* p* = 0.96, *d* = 0.36). Likewise, in primiparous rats tested during metestrus, relative to vehicle, both the low and high doses of diazepam increased the time spent in the open arms and increased number of open-arm entries (*ps* < 0.01,* d*s > 2.0), but had no effect on the number of closed-arm entries (smallest *p* = 0.28,* d* = 1.58).

In the “anxiety index”, there was a main effect of drug (*F*_(2,44)_ = 29.67, *p* < 0.001, ηp2 = 0.57) but no main effect of estrous phase or estrous × drug interaction (*F*s < 1). In primiparous rats tested during proestrus, relative to vehicle, both the low and high doses of diazepam decreased the anxiety index (*ps* < 0.01,* d*s > 2.0). Likewise, in primiparous rats tested during metestrus, relative to vehicle, both the low and high doses of diazepam decreased the anxiety index (*ps* < 0.01,* d*s > 2.0).

### Experiment 2: Does reproductive experience alter the effects of chronic fluoxetine on fear conditioning/extinction, anxiety-like behaviour, and corticosterone levels?

#### Fear conditioning and extinction

##### Fear conditioning

**Fig. 3:**
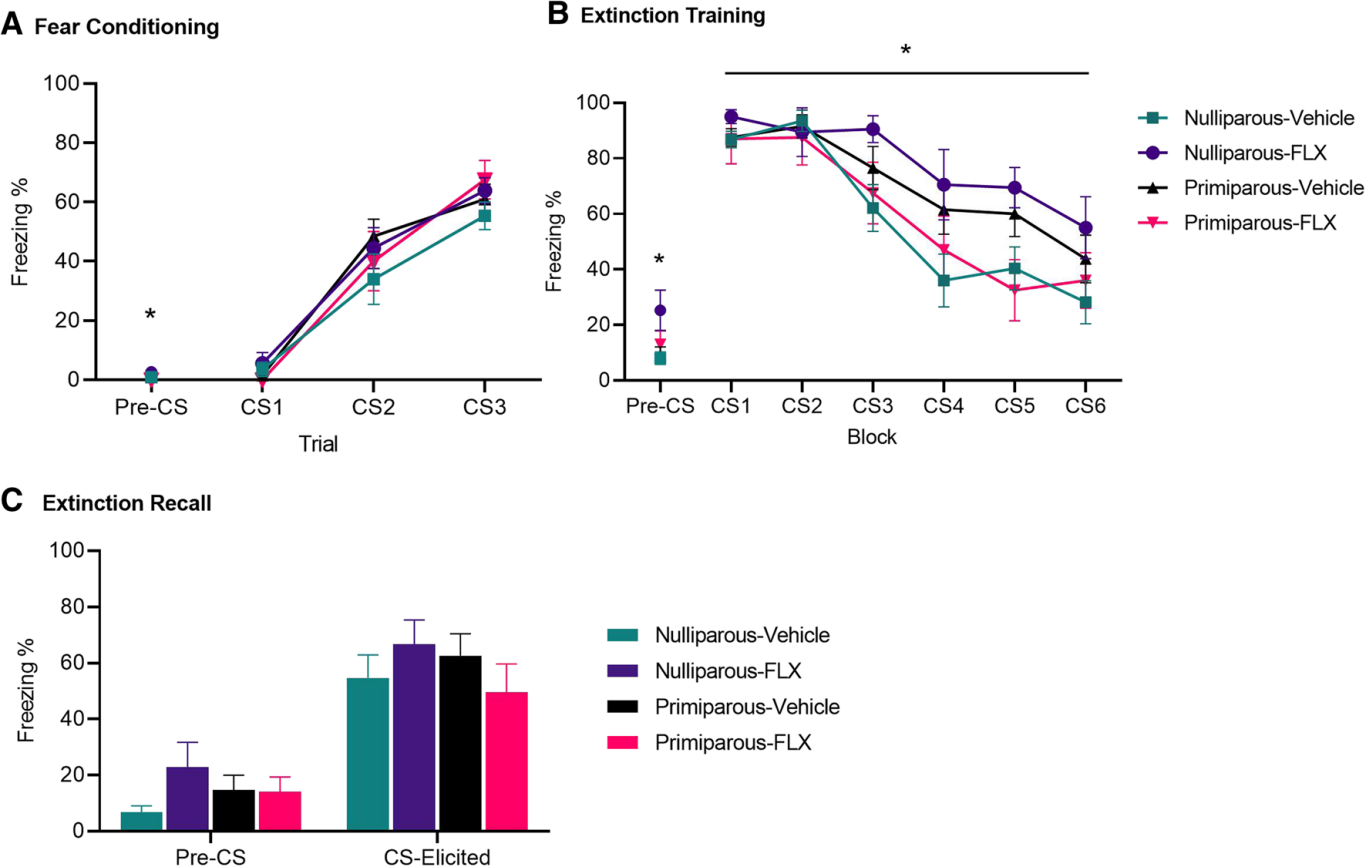
Nulliparous and primiparous rats administered either chronic fluoxetine or vehicle underwent fear conditioning, fear extinction during metestrus, and were tested for extinction recall. **A** Mean (± SEM) levels of pre-CS and CS-elicited freezing for groups nulliparous-vehicle (*n* = 14), nulliparous-fluoxetine (*n* = 9), primiparous-vehicle (*n* = 16), and primiparous-fluoxetine (*n* = 10) during fear conditioning. *Primiparous-vehicle > primiparous-fluoxetine in pre-CS freezing (*p* < .05). **B** Mean (± SEM) pre-CS and CS-elicited freezing for groups during extinction training. The data are presented as 6 blocks of trials, each representing an average of five trials. *Fluoxetine groups > vehicle groups in pre-CS freezing (*p* < .05). *Nulliparous-fluoxetine > nulliparous-vehicle on block 3, 4, 5 (ps < .05). **C** Mean (± SEM) pre-CS and CS-elicited freezing for groups during extinction recall. Note: FLX fluoxetine

There were no main effects of drug or reproductive status on baseline freezing (see Fig. 3A, largest *F*_(1,45)_ = 2.10, *p* = 0.15, ηp2 = 0.05) but there was a drug × reproductive status interaction (*F*_(1,45)_ = 4.84,* p* = . 03, ηp2 = 0.10). Follow-up *t-*tests revealed that primiparous-fluoxetine rats displayed lower pre-CS freezing compared to primiparous-vehicle rats (*t*_(24)_ = 2.33, *p* = 0.03, *d* = 0.74), whereas nulliparous-fluoxetine and nulliparous-vehicle groups did not differ (t_(21)_ = 1.37,* p* = 0.18, *d* = 0.59). CS-elicited freezing increased across fear conditioning (effect of conditioning trial; *F*_(2,90)_ = 121.40, *p* < 0.001, ηp2 = 0.73) similarly in all groups (no main effects of reproductive status or drug, or interaction between factors, and no interaction between these factors and conditioning trial, largest* F*_(1,45)_ = 1.26, *p* = 0.27, ηp2 = 0.03). Baseline freezing was entered as covariate but the results remained the same (no main effects or interactions; largest *F*_(1,44)_ = 1.30, *p* = 0.26).

##### Extinction training

There were no main effects of reproductive status or drug × reproductive status interaction on baseline freezing (see Fig. 2B, largest *F*_(1,45)_ = 2.64,* p* = 0.11, ηp2 = 0.06) but there was a main effect of drug whereby rats receiving fluoxetine had significantly higher freezing compared to those receiving vehicle (*F*_(1,45)_ = 6.43, *p* = 0.02, ηp2 = 0.13). This main effect was primarily driven by nulliparous groups. Post hoc *t-*tests revealed that nulliparous-fluoxetine rats had higher pre-CS freezing compared to nulliparous-vehicle rats (*t*_(21)_ = 2.69, *p* = 0.01, *d* = 1.15), whereas primiparous-fluoxetine and primiparous-vehicle rats did not differ (*t* < 1). As expected, CS-elicited freezing decreased across extinction training (effect of extinction block; (*F*_(5,225)_ = 43.04, *p* < 0.001, ηp2 = 0.49). There were no main effects of reproductive status or drug (*F*s < 1), but there was a reproductive status × drug interaction (*F*_(1,45)_ = 7.47, *p* = 0.01, ηp2 = 14), and a reproductive status × drug × extinction block interaction (*F*_(5,225)_ = 3.02*, p* = 0.02, ηp2 = 0.06). Follow-up *t-*tests revealed that nulliparous-fluoxetine rats had higher CS-elicited freezing than nulliparous-vehicle rats on block 3 (*t*_(21)_ = 2.52, *p* = 0.01, *d* = 1.08), block 4 (*t*_(21)_ = 2.21, *p* = 0.04, *d* = 0.94), and block 5 (*t*_(21)_ = 2.56, *p* = 0.02, *d* = 1.09); the difference for block 6 did not reach significance (*t*_(21)_ = 2.03, *p* = 0.055, *d* = 0.87). In contrast, primiparous-fluoxetine rats exhibited lower CS-elicited freezing compared to primiparous-vehicle rats during block 5 but this did not reach significance (*t*_(24)_ = 2.00, *p* = 0.057, *d* = 0.81), and comparable CS-elicited freezing on all other blocks (*t*s < 1). Baseline freezing was entered as covariate but the results remained similar (reproductive status × drug interaction (*F*_(1,44)_ = 5.30, *p* = 0.03); however, the reproductive status × drug × extinction block interaction was reduced to a trend (*F*_(5, 220)_ = 2.30, *p* = 0.06)).

##### Extinction recall

All groups showed comparable baseline freezing (see Fig. 3C, no main effects of estrous phase or drug and no interaction between factors; largest *F*_(1,45)_ = 2.34, *p* = 0.13, ηp2 = 0.05). During extinction recall, there were no group differences in CS-elicited freezing (no main effects or interaction; largest *F*_(1,45)_ = 2.01, *p* = 0.16, ηp2 = 0.04). Nulliparous-vehicle and nulliparous-fluoxetine rats showed comparable CS-elicited freezing at extinction recall (*t*_(21)_ = 0.97, *p* = 0.34, *d* = 0.44), as did primiparous-vehicle and primiparous-fluoxetine rats (*t*_(24)_ = 1.04, *p* = 0.31, *d* = 0.42).

#### Elevated plus maze

**Fig. 4:**
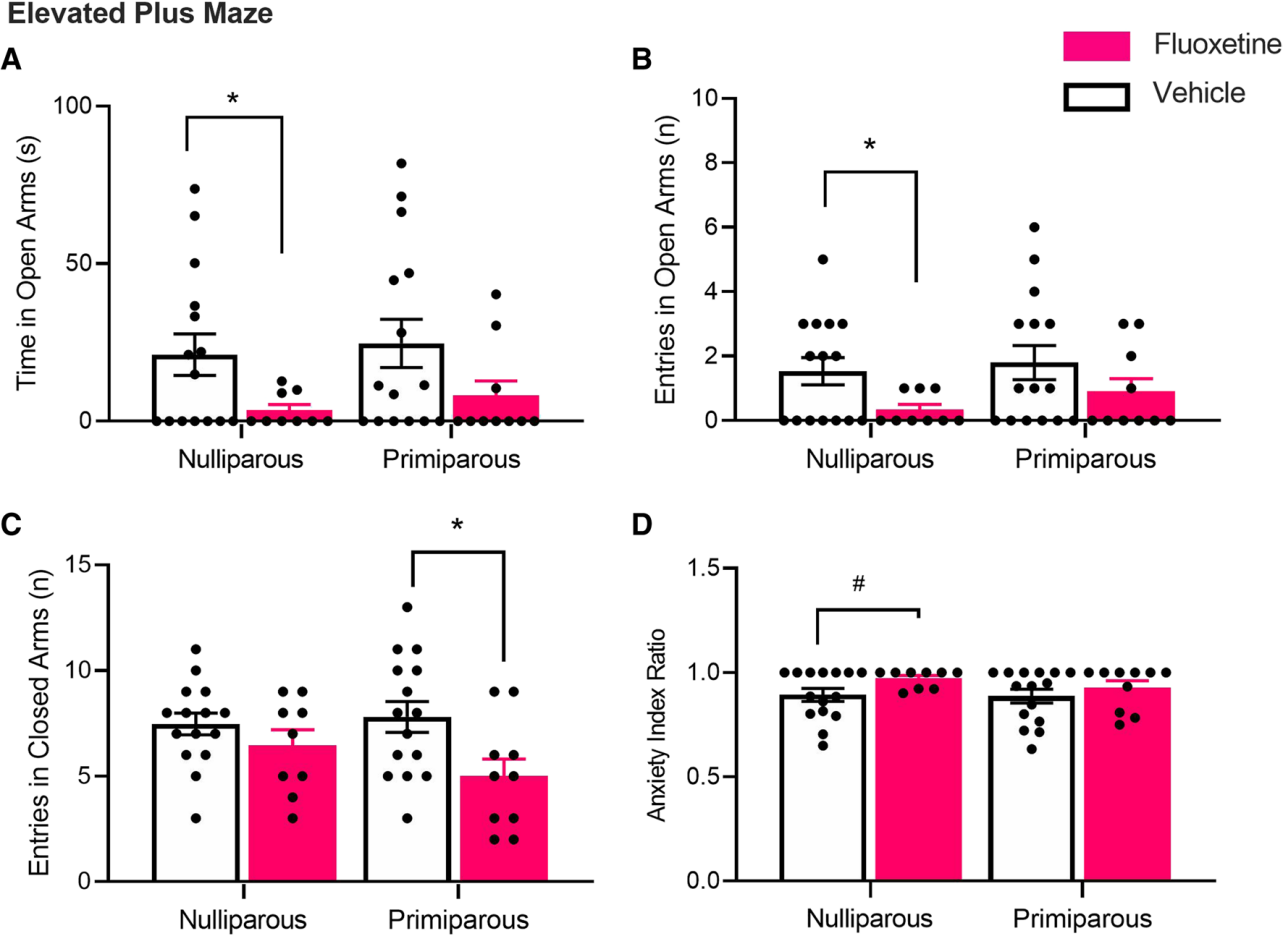
Nulliparous and primiparous rats administered either chronic fluoxetine or vehicle were tested on the elevated plus maze for 5 min. **A** Mean (± SEM) time spent in the open arms in groups nulliparous-vehicle (*n* = 15), nulliparous-fluoxetine (*n* = 9), primiparous-vehicle (*n* = 15), and primiparous-fluoxetine (*n* = 10). *Nulliparous-vehicle > nulliparous-fluoxetine (*p* < .025). **B** Mean (± SEM) number of entries in the open arms. *Nulliparous-vehicle > nulliparous-fluoxetine (*p* < .025). **C** Mean (± SEM) number of entries in the closed arms. *Primiparous-vehicle > primiparous-fluoxetine (*p* < .025). **D** Mean (± SEM) anxiety index score. #Nulliparous-vehicle < nulliparous-fluoxetine (*p* < .05)

Figure 4 depicts means (± SEM) for behavioural measures on the EPM in nulliparous and primiparous rats. There were no main effects of reproductive status or reproductive status × drug interactions (*F*s < 1), but there were main effects of drug in the time spent in the open arms (*F*_(1,45)_ = 6.46, *p* = 0.02, ηp2 = 0.13) and number of open-arm entries (*F*_(1,45)_ = 5.10, *p* = 0.03, ηp2 = 0.10). These main effects were driven by nulliparous rats, as nulliparous-fluoxetine rats spent less time (*t*_(22)_ = 2.58, *p* = 0.02, *d* = 0.86) and made less entries (*t*_(22)_ = 2.64, *p* = 0.02, *d* = 0.89) in the open arms than nulliparous-vehicle rats, whereas primiparous-fluoxetine rats and primiparous-vehicle rats did not differ in the time spent (*t*_(23)_ = 1.85, *p* = 0.08, *d* = 0.67) or entries (*t*_(23)_ = 1.24, *p* = 0.23, *d* = 0.50) in the open arms. In the number of closed-arm entries, there was no main effect of reproductive status or reproductive status × drug interaction (largest *F*_(1,45)_ = 1.54, *p* = 0.22), but there was a main effect of drug (*F*_(1,45)_ = 7.11, *p* = 0.01, ηp2 = 0.14). This was primarily driven by primiparous rats as primiparous-fluoxetine rats made smaller number of closed-arm entries than primiparous-vehicle rats (*t*_(23)_ = 2.51, *p* = 0.02, *d* = 1.02), whereas nulliparous-fluoxetine rats and nulliparous-vehicle rats did not differ (*t*_(22)_ = 1.16, *p* = 0.26, *d* = 0.49). In the “anxiety index”, there were no main effects or interaction (largest *F*_(1,45)_ = 3.42, *p* = 0.07, ηp2 = 0.07). Nulliparous-fluoxetine rats tended to have a higher anxiety index score than nulliparous-vehicle rats; however, this effect did not withstand Bonferroni corrections (*t*_(22)_ = 2.32, *p* = 0.03, *d* = 0.80). Primiparous-fluoxetine and primiparous-vehicle rats did not differ (*t*_(23)_ = 0.83, *p* = 0.40, *d* = *0.3*4).

#### Plasma corticosterone

**Fig. 5:**
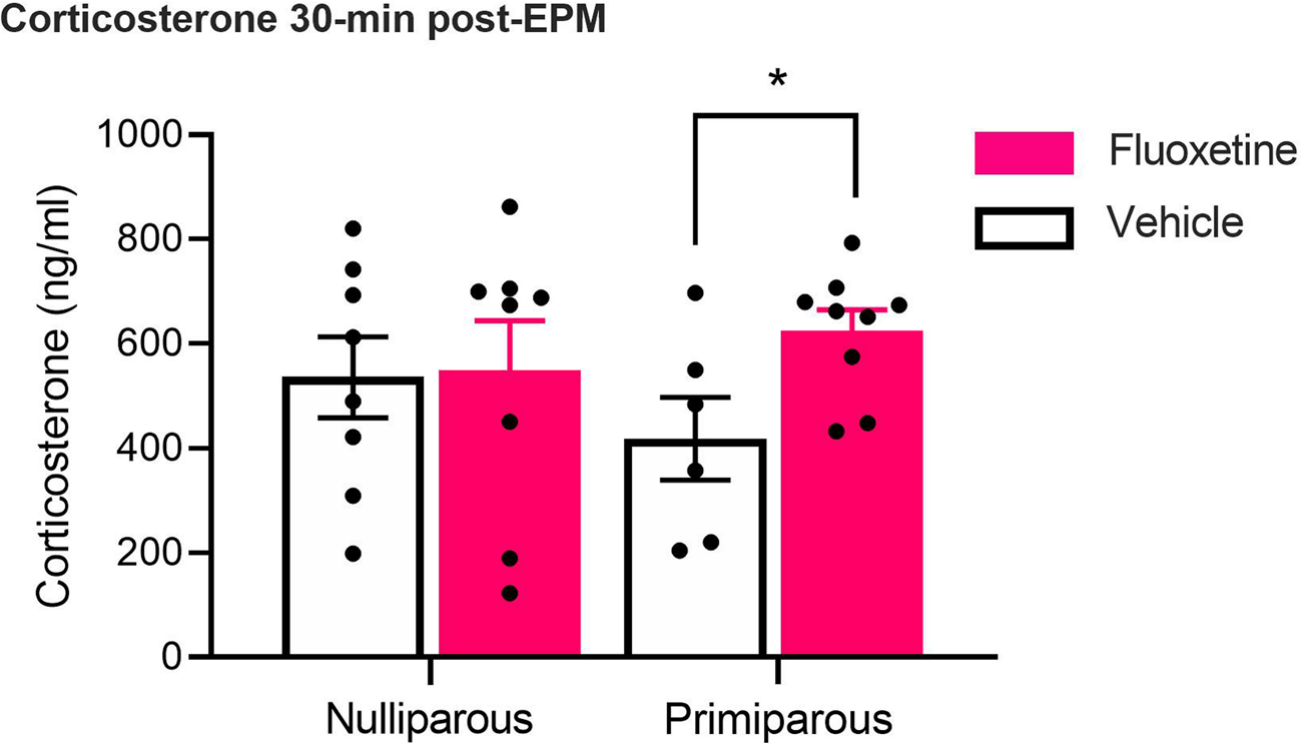
Nulliparous and primiparous rats administered either chronic fluoxetine or vehicle were euthanised 30 min after the initiation of the elevated plus maze, and plasma in trunk blood was rapidly extracted. Mean (± SEM) corticosterone levels in plasma for nulliparous-vehicle (*n* = 8), nulliparous-fluoxetine (*n* = 8), primiparous-vehicle (*n* = 6), and primiparous-fluoxetine (*n* = 9). *Primiparous-fluoxetine > primiparous-vehicle (*p* < .025)

There were no main effects of drug or reproductive status, and no interaction between factors (see Fig. 5; largest *F*_(1,27)_ = 2.16,* p* = 0.15, ηp2 = 0.07). Nulliparous-fluoxetine rats and nulliparous-vehicle rats showed comparable levels of plasma corticosterone (*t*_(14)_ = 0.11, *p* = 0.92,* d* = 0.05). In contrast, primiparous-fluoxetine rats had higher levels of plasma corticosterone compared to primiparous-vehicle rats (*t*_(13)_ = 2.57, *p* = 0.02,* d* = 1.35). Pearson’s bivariate correlations revealed no correlation between corticosterone concentration and behavioural measures on the EPM (data not shown; largest *r* =  − 0.07, *p* = 0.72).

#### Body weight

During the first 2 weeks of drug administration, chronic fluoxetine caused a reduction in body weight in nulliparous females but had no effect on body weight in primiparous females (see Supplemental Information for analysis, results, and discussion).

## Discussion

The current study found that reproductive experience alters the drug response to two commonly used treatments for anxiety, diazepam and fluoxetine, in female rats. Consistent with previous research (Fernandez-Guasti and Picazo 1990; Molina-Hernandez et al. 2001, 2013; Soares-Rachetti Vde et al. 2016), in Experiment 1, we found that both doses of diazepam produced anxiolytic-like effects in nulliparous rats during proestrus, whereas the higher, but not the lower dose of diazepam produced anxiolytic-like effects during metestrus. These effects cannot be attributed to the sedative-like effects of diazepam on general locomotor activity given that diazepam had little effect on number of closed-arm entries, and that the anxiety index accounts for potential drug-induced changes in general locomotor activity. Moreover, these effects cannot be due to estrous effects on basal anxiety-like behaviour given that vehicle-treated rats showed comparable anxiety-like behaviour in proestrus and metestrus. Previous studies have reported mixed findings on estrous effects on the EPM (Lovick and Zangrossi 2021; Pestana et al. 2022), and we recently reported that estrous effects on anxiety-like behaviour are mitigated in primiparous rats (Pestana et al. 2023). One potential reason for why we did not detect estrous effects on the EPM in vehicle-treated nulliparous rats is because we used a between-subjects design in the current study, as opposed to the within-subjects design in our previous study (Pestana et al. 2023). Irrespective, the novel finding of Experiment 1 was that both the low and high dose of diazepam produced anxiolytic-like effects during proestrus and metestrus in primiparous rats, in contrast to the findings in nulliparous rats. The finding that the lower dose of diazepam produced anxiolytic-like effects during metestrus in primiparous, but not nulliparous rats, is consistent with Byrnes and Bridges (2006), who found that primiparous rats have a higher sensitivity to the anxiolytic-like effects of 2.0 mg/kg of diazepam compared to nulliparous rats. Taken together, the results of Experiment 1 suggest that reproductive experience may mitigate the influence of estrous cycle on the sensitivity to diazepam, and increase the sensitivity to lower doses of diazepam during metestrus, effects that persist for at least 1-month post-weaning when the hormonal surges of pregnancy and lactation have diminished. These findings are conceptually similar to our previous findings in anxiety-like behaviour and fear extinction, which are estrous cycle dependent in nulliparous but not primiparous rats (Milligan-Saville and Graham 2016; Pestana et al. 2023, 2021; Tang and Graham 2020). In contrast, reproductive experience does not alter estrous effects on non-emotional tasks, such as the place- and object-recognition memory tasks (Paris and Frye 2008). Together, these data indicate that reproductive experience does not have a global impact on estrous effects but rather that reproductive experience mitigates estrous effects on certain tasks specifically related to anxiety regulation, such as fear extinction, anxiety-like behaviour, and diazepam sensitivity.

Reproductive experience causes an array of long-term neurobiological and physiological changes that could mitigate estrous effects on diazepam sensitivity. In nulliparous rats, increases in allopregnanolone levels during proestrus are associated with heightened sensitivity to diazepam, but the subsequent *rapid decline* in allopregnanolone reduces the sensitivity to diazepam during metestrus/diestrus, potentially by upregulating the expression of GABA_A_R subunits that render the receptor insensitive to benzodiazepines (Lovick et al. 2005; Soares-Rachetti Vde et al. 2016). Compared to nulliparous rats, primiparous rats have a lower peak in allopregnanolone during the afternoon of proestrus, and a subsequent blunted and non-significant decline during the evening of proestrus (Pestana et al. 2023). As such, one possible account for why primiparous females exhibit estrous cycle–*independent* diazepam sensitivity is that the *gradual decline* in allopregnanolone during the evening of proestrus prevents withdrawal-like effects during metestrus. Reproductive experience also reduces levels of circulating estradiol in young adult female rats during proestrus (Bridges and Byrnes 2006; Milligan-Saville and Graham 2016). Estradiol modulates GABA_A_R expression (Daendee et al. 2013; Herbison and Fénelon 1995) and the sensitivity to diazepam in nulliparous rats (Nomikos and Spyraki 1988). As such, another possibility is that the reduced levels of estradiol during proestrus may, in part, blunt estrous effects on the sensitivity to diazepam in primiparous rats. In addition to changes in ovarian steroid levels, reproductive experience causes long-term changes in GABA_A_R subunit expression in brain regions that benzodiazepines target including the amygdala and hippocampus (Byrnes et al. 2007; Pestana et al. 2023; Heldt and Ressler 2006). For example, we found that, compared to nulliparous rats, primiparous rats had increased expression of all GABA_A_R subunits examined in the ventral hippocampus, including benzodiazepine-sensitive subunits from the α family (α1, α2, α5), a benzodiazepine-insensitive subunit (α4), and the ß2 subunit from the ß family (Pestana et al. 2023). Therefore, one may speculate that reproductive experience upregulates the number of GABA_A_Rs in the ventral hippocampus, which in turn could alter the sensitivity to diazepam in primiparous rats as there are more receptors in the hippocampus to which diazepam can bind. Future studies are required to investigate whether the motherhood-induced changes in ovarian steroid levels and GABA_A_R subunit expression are causally related to the altered sensitivity to diazepam across the estrous cycle in primiparous rats.

In addition to diazepam, in Experiment 2, we found that the effects of fluoxetine on behavioural and hormonal indices of fear and anxiety in female rats differ depending on their reproductive history. That is, in nulliparous rats, fluoxetine increased freezing during the latter blocks of extinction training but had no effect on fear conditioning or extinction recall, whereas fluoxetine had no impact on conditioning or extinction in primiparous rats. Given that fluoxetine was administered prior to fear conditioning and extinction training, it is difficult to determine whether the drug effects on extinction training in nulliparous rats were due to its anxiogenic-like properties or impairments in fear learning and/or memory processes. Consistent with previous research (Gray and Hughes 2015; Pawluski et al. 2012), fluoxetine produced anxiogenic-like effects in nulliparous rats, evidenced by a reduction in the number of open-arm entries, time spent in the open arms, and a trend for an increased anxiety index on the EPM. These effects cannot be attributed to the sedative-like effects of fluoxetine on general locomotor activity given that chronic fluoxetine had no effect on the number of closed-arm entries, and also tended to increase the anxiety index which accounts for potential differences in general locomotor activity. Although the pattern of results was in the same direction, fluoxetine had no significant effect on anxiety-like behaviour in primiparous females. This null effect was inconsistent with Pawluski et al. (2012) who found that chronic fluoxetine (5 mg/kg) produced anxiogenic-like effects on the elevated zero maze in primiparous rats a few days post-weaning. Together, these findings may suggest that an increased statistical power may be required to detect anxiogenic-like effects on the EPM in primiparous rats. Regardless, one possible account for why fluoxetine increased freezing during extinction training in nulliparous, but not primiparous females, is due to the anxiogenic-like effects of the drug that were detected in nulliparous rats only.

It is also possible that chronic fluoxetine may have increased freezing during extinction training in nulliparous but not primiparous rats due to impairments in fear learning and/or memory processes. For instance, as chronic fluoxetine was administered prior to fear conditioning, it is possible that fluoxetine impacted the acquisition and/or consolidation of fear conditioning in nulliparous but not primiparous rats. However, chronic fluoxetine had no effect on freezing levels during fear conditioning in nulliparous rats, suggesting that fluoxetine- and vehicle-treated rats acquired comparable fear conditioning. In addition, chronic fluoxetine had no effect on the first two blocks of extinction training despite increasing freezing levels during later extinction blocks, which may suggest that chronic fluoxetine had little impact on the consolidation of the conditioning memory. However, ceiling effects during the first two blocks of extinction training may have prevented the ability to detect differences between fluoxetine- and vehicle-treated rats during early extinction training. In addition to impacting fear conditioning, it is also possible that chronic fluoxetine impaired the learning and/or memory processes underlying fear extinction. For example, the increased freezing levels during the later, but not early, blocks of extinction training in nulliparous rats may reflect impairments in the acquisition of extinction. While the current results are inconsistent with Lebrón-Milad et al. (2013) who found that chronic fluoxetine reduced freezing during extinction training and extinction recall in nulliparous rats, the current findings are consistent with Burghardt et al. (2013) who found that chronic citalopram (10 mg/kg), another commonly prescribed SSRI, increased freezing levels in the later, but not early, blocks of extinction training in male rats. However, Burghardt et al. (2013) also found that chronic citalopram impaired extinction recall in male rats, whereas chronic fluoxetine had no effect on extinction recall in nulliparous rats in the current study. Given that nulliparous rats were extinguished during metestrus when extinction recall is typically impaired, ceiling effects may have obscured the detection of an impairing effect of fluoxetine on extinction recall. To determine whether chronic fluoxetine impairs extinction recall in nulliparous rats, future studies should extinguish nulliparous rats during proestrus when they do not show impaired extinction recall. Investigating this possibility is of interest as the estrous cycle impacted the effects of fluoxetine on fear extinction in nulliparous rats (Lebrón-Milad et al. 2013).

If chronic fluoxetine increases freezing during extinction training in nulliparous rats by impairing the acquisition of extinction, then one possible account for why chronic fluoxetine had no effect on extinction training in primiparous rats is due to the distinct neurobiological and behavioural features of fear extinction following reproductive experience. For instance, Burghardt et al. (2013) found that chronic citalopram downregulated protein expression of the NMDAR subunit NR2B in the lateral amygdala, and that NR2B protein levels were negatively correlated with freezing levels exhibited by male rats during extinction training. Burghardt et al. (2013) proposed that chronic citalopram impaired the acquisition of extinction via the downregulation of NMDARs within the amygdala. Unlike nulliparous female and male rats, fear extinction appears to be independent of NMDARs in primiparous females (Tang and Graham 2019a, b). As such, one possibility is that chronic fluoxetine impaired the acquisition of extinction in nulliparous rats via the modulation of NMDARs but had no effect in primiparous females because they do not require NMDARs for fear extinction. Future studies are required to investigate this possibility. Future studies should also investigate other behavioural measures of fear in addition to freezing (e.g. darting) in females as there may be sex-specific conditioned fear behaviours (Gruene et al. 2015).

In contrast to the behavioural findings, chronic fluoxetine increased plasma corticosterone 30-min post-EPM in primiparous but not nulliparous females. The dissociation between the behavioural and hormonal effects of fluoxetine in nulliparous versus primiparous rats is conceptually consistent with previous research showing that anxiety-like behaviour is not associated with corticosterone secretion post-EPM in male or female rats (Mikics et al. 2005; Neumann et al. 1998; Rodgers et al. 1999). Moreover, the finding that chronic fluoxetine increased plasma corticosterone in primiparous but not nulliparous rats is inconsistent with Workman et al. (2016), who found that chronic fluoxetine lowered plasma corticosterone 90 min after the forced swim test in nulliparous rats but had no effect in primiparous rats a few days post-weaning. However, corticosterone secretion following an acute stressor may be influenced by several factors, including the type of stressor, timepoint of collection since the stressor occurred, and the time of collection relative to weaning in primiparous rats. In addition, the current study is limited in that we did measure basal corticosterone levels. As such, it cannot be determined whether the impact of fluoxetine on corticosterone levels in primiparous rats was due to increasing the stress-induced corticosterone levels, or whether this was a consequence of altering basal corticosterone levels. Another limitation of the current study is that we only tested one dose of fluoxetine (10 mg/kg). As such, it cannot be determined whether reproductive experience alters the sensitivity to fluoxetine.

In conclusion, the current findings that reproductive experience alters the responsivity to diazepam and chronic fluoxetine in female rats adds to a growing body of work illustrating the importance of considering the impact of sex-specific factors, such as estrous cycle and reproductive status, on animal studies examining the pharmacological treatment of anxiety disorders. Yet, single-sex studies on pharmacological treatments in male rodents outnumber those in female rodents 5 to 1 (Beery and Zucker 2011), and only a handful of studies have examined reproductively experienced female rodents. Investigating the drug response in female rodents with and without reproductive experience may lead to existing medications being better tailored and effective for women across their entire lifespan.

### Supplementary Information

Below is the link to the electronic supplementary material.

Supplementary file1 (DOCX 90.2 KB)

## Data Availability

Data is available from the authors upon request.
