# Supplementary material for: Reproductive experience alters the effects of diazepam and fluoxetine on anxiety-like behaviour, fear extinction, and corticosterone levels in female rats
Source: Psychopharmacology (Berl). 2023 Aug 15;240(12):2515–28. doi: 10.1007/s00213-023-06446-z (PMC10640474; doi:10.1007/s00213-023-06446-z)
Supplement: Supplementary file 1 — Supplementary file1 (DOCX 90.2 KB) [file 213_2023_6446_MOESM1_ESM.docx]

**Reproductive experience alters the effects of diazepam and fluoxetine on anxiety-like behaviour, fear extinction, and corticosterone levels in female rats**

***Supplemental Information***

**Supplemental Methods and Materials - Experiments Reported in Manuscript**

***Experiment 2 – Drug Administration***

Fluoxetine hydrochloride powder was dissolved in tap water at a daily dose of approximately 10mg/kg. The vehicle was tap water. Fluoxetine and vehicle were made available ad libitum in their drinking water in bottles covered with aluminium foil to protect the drug from light, as per Graham, Dong, and Richardson (2018). Given that fluoxetine is relatively stable when dissolved in water, the drinking water containing the drug was changed every two days. Rats in boxes receiving fluoxetine were weighed every second day. The drinking bottles in their box were also weighed to determine the amount of fluid consumed over the two-day period. The appropriate amount of fluoxetine powder to be administered was calculated based upon the average weight of the rats in each box. The fluoxetine powder was then mixed in the same amount of water that was consumed over the previous two days in each box. This procedure ensured that rats were administered a stable dose of fluoxetine despite variations in the amount of fluid consumed between boxes or across the experiment. That is, if one box of rats drank a smaller amount of water over the last two days, fluoxetine would be administered in a smaller amount of water for the next two days, meaning that these rats would still ingest the appropriate dose of fluoxetine, albeit with slight variations in the fluoxetine to water ratio. Although this procedure meant that there were slight variations in the actual dose of fluoxetine received by each individual rat, depending on how much water they drank, this method was preferable to individually housing animals or delivering fluoxetine chronically via daily injections as these procedures would be highly stressful for the animals and potentially confound measures of conditioned fear and anxiety-like behaviour. Moreover, administrating SSRIs to animals via their drinking water is a common practice having been conducted numerous times in the literature (Graham et al., 2018; Gunduz-Cinar et al., 2016; Karpova et al., 2011; Lebrón-Milad et al., 2013; Norcross et al., 2008). The total volume of water in which the fluoxetine was dissolved was adjusted as necessary so that the amount of water remaining in the bottles after 2 days was between 50 and 100 mL. This ensured that the rats had sufficient drinking water while still receiving the correct dose of fluoxetine. To control for any potential effects of handling on behaviour, vehicle-treated rats and their drinking bottles were weighed on the same days as those receiving fluoxetine. The drug administration procedure continued until the end of the experiment, once all rats in the box had been euthanised. Given that rats were euthanised 30 min after the initiation of the EPM, this meant that the number of rats in each box was continually reduced throughout the experiment. When this occurred, the total amount of fluoxetine was adjusted to the appropriate number of rats in the box

**Supplemental Results**

***Experiment 2 - Body weight during the drug administration period***

A mixed model ANOVA with the between-subjects of reproductive status (nulliparous or primiparous) and drug (fluoxetine or vehicle) and the within-subjects factor of time (Days 1, 3, 5, 7, 9, 11, 13) was used to analyse body weight during the two-week drug administration period. Body weight decreased across the two week administration period prior to behavioural testing (significant effect of day; *F*_(6,282)_=29.52, *p*<.001; see **Fig. S1**). There was no significant main effect of drug or reproductive status, and no significant interaction between factors (largest *F*_(1,47)_=1.87, *p=*.18). However, there was a significant day x drug interaction (*F*_(6,282)_=47.54, <.001), significant day x reproductive status interaction (*F*_(6,282)_=12.78, *p*<.001) and a significant day x drug x reproductive status interaction (*F*_(6,282)_=6.62, *p*<.001). Follow up *t-*tests revealed that nulliparous-fluoxetine rats had significantly lower weight compared to nulliparous-vehicle rats on day 9 (*t*_(23)_=2.12, *p* .045, *d=*.86), as well as day 11 (*t*_(23)_=2.05, *p=*.052, *d*=.84) and day 13 (*t*_(23)_=1.90, *p=*.07, *d=*.78) but these effects did not reach significance. In contrast, primiparous-fluoxetine rats and primiparous-vehicle rats showed comparable body weight on all days (largest *t*_(24)_=1.01, *p*=.32, *d*=.40).

These findings suggest that chronic fluoxetine led to weight loss in nulliparous rats but had no effect in primiparous rats. The finding that chronic fluoxetine led to weight loss in nulliparous rats is consistent with studies showing that sub-chronic and chronic administration of fluoxetine reduces body weight in male rats using various modes of delivery, such as intraperitoneal injection (Damjanoska et al., 2003; Horowitz et al., 2003; Perrone et al., 2004). In addition, meta-analyses in humans have shown that weight loss is a common side effect of fluoxetine treatment (Domecq et al., 2015; Serretti & Mandelli, 2010).Together, these findings suggest that the loss of body weight in nulliparous rats was due to the pharmacological properties of the drug, rather than the oral mode of delivery (e.g., due to fluoxetine altering the taste of the water which reduced water and food consumption). In addition, similar to the current experiment, Workman et al. (2016) found that chronic fluoxetine reduced body weight in nulliparous but not primiparous rats during the postpartum period (i.e., a few days post-weaning). Together, these findings suggest that the effects of chronic fluoxetine on body weight dynamics may be altered in females following reproductive experience. In addition, it is possible that reproductive experience alters the metabolism of fluoxetine, although to our knowledge, no studies have assessed this possibility.

**Fig. S1.** Nulliparous and primiparous rats were administered fluoxetine or vehicle via their drinking water for two weeks prior to behavioural testing, with rats being weighed every second day prior to changing the water. **(A)** Mean (±SEM) body weight of groups Nulliparous-Vehicle (n = 15), Nulliparous-Fluoxetine (n = 10), Primiparous-Vehicle (n = 16), Primiparous-Fluoxetine (n = 10). *Nulliparous-Fluoxetine < Nulliparous Proestrus on day 9 (p < .05).

**Supplemental References**

Damjanoska, K. J., Van de Kar, L. D., Kindel, G. H., Zhang, Y., D'Souza, D. N., Garcia, F., . . . Muma, N. A. (2003). Chronic fluoxetine differentially affects 5-hydroxytryptamine (2A) receptor signaling in frontal cortex, oxytocin- and corticotropin-releasing factor-containing neurons in rat paraventricular nucleus. *J Pharmacol Exp Ther*, *306*(2), 563-571. <https://doi.org/10.1124/jpet.103.050534>

Domecq, J. P., Prutsky, G., Leppin, A., Sonbol, M. B., Altayar, O., Undavalli, C., . . . Murad, M. H. (2015). Clinical review: Drugs commonly associated with weight change: a systematic review and meta-analysis. *J Clin Endocrinol Metab*, *100*(2), 363-370. <https://doi.org/10.1210/jc.2014-3421>

Graham, B. M., Dong, V., & Richardson, R. (2018). The impact of chronic fluoxetine on conditioned fear expression and hippocampal FGF2 in rats: Short- and long-term effects. *Neurobiol Learn Mem*, *155*, 344-350. <https://doi.org/10.1016/j.nlm.2018.09.004>

Gunduz-Cinar, O., Flynn, S., Brockway, E., Kaugars, K., Baldi, R., Ramikie, T. S., . . . Holmes, A. (2016). Fluoxetine Facilitates Fear Extinction Through Amygdala Endocannabinoids. *Neuropsychopharmacology*, *41*(6), 1598-1609. <https://doi.org/10.1038/npp.2015.318>

Horowitz, J. M., Goyal, A., Ramdeen, N., Hallas, B. H., Horowitz, A. T., & Torres, G. (2003). Characterization of fluoxetine plus olanzapine treatment in rats: a behavior, endocrine, and immediate-early gene expression analysis. *Synapse*, *50*(4), 353-364. <https://doi.org/10.1002/syn.10276>

Karpova, N. N., Pickenhagen, A., Lindholm, J., Tiraboschi, E., Kulesskaya, N., Agústsdóttir, A., . . . Castrén, E. (2011). Fear erasure in mice requires synergy between antidepressant drugs and extinction training. *Science*, *334*(6063), 1731-1734. <https://doi.org/10.1126/science.1214592>

Lebrón-Milad, K., Tsareva, A., Ahmed, N., & Milad, M. R. (2013). Sex differences and estrous cycle in female rats interact with the effects of fluoxetine treatment on fear extinction. *Behav Brain Res*, *253*, 217-222. <https://doi.org/10.1016/j.bbr.2013.07.024>

Norcross, M., Mathur, P., Enoch, A. J., Karlsson, R. M., Brigman, J. L., Cameron, H. A., . . . Holmes, A. (2008). Effects of adolescent fluoxetine treatment on fear-, anxiety- or stress-related behaviors in C57BL/6J or BALB/cJ mice. *Psychopharmacology (Berl)*, *200*(3), 413-424. <https://doi.org/10.1007/s00213-008-1215-7>

Perrone, J. A., Chabla, J. M., Hallas, B. H., Horowitz, J. M., & Torres, G. (2004). Weight loss dynamics during combined fluoxetine and olanzapine treatment. *BMC Pharmacol*, *4*, 27. <https://doi.org/10.1186/1471-2210-4-27>

Serretti, A., & Mandelli, L. (2010). Antidepressants and body weight: a comprehensive review and meta-analysis. *J Clin Psychiatry*, *71*(10), 1259-1272. <https://doi.org/10.4088/JCP.09r05346blu>

Workman, J. L., Gobinath, A. R., Kitay, N. F., Chow, C., Brummelte, S., & Galea, L. A. M. (2016). Parity modifies the effects of fluoxetine and corticosterone on behavior, stress reactivity, and hippocampal neurogenesis. *Neuropharmacology*, *105*, 443-453. <https://doi.org/10.1016/j.neuropharm.2015.11.027>
